# Reduced EGFR and increased miR-221 is associated with increased resistance to temozolomide and radiotherapy in glioblastoma

**DOI:** 10.1038/s41598-020-74746-x

**Published:** 2020-10-20

**Authors:** Zammam Areeb, Sarah F. Stuart, Alice J. West, Juliana Gomez, Hong P. T. Nguyen, Lucia Paradiso, Ahmad Zulkifli, Jordan Jones, Andrew H. Kaye, Andrew P. Morokoff, Rodney B. Luwor

**Affiliations:** 1 Level 5, Clinical Sciences Building, Department of Surgery, The University of Melbourne, The Royal Melbourne Hospital, Parkville, VIC 3050 Australia; 2 Department of Neurosurgery, The Royal Melbourne Hospital, Parkville, VIC 3050 Australia; 3 Department of Neurosurgery, Hadassah Hebrew University Medical Centre, 91120 Jerusalem, Israel

**Keywords:** Oncogenes, Cell signalling, Cancer therapeutic resistance

## Abstract

Despite aggressive treatment with temozolomide and radiotherapy and extensive research into alternative therapies there has been little improvement in Glioblastoma patient survival. Median survival time remains between 12 and 15 months mainly due to treatment resistance and tumor recurrence. In this study, we aimed to explore the underlying mechanisms behind treatment resistance and the lack of success with anti-EGFR therapy in the clinic. After generating a number of treatment resistant Glioblastoma cell lines we observed that resistant cell lines lacked EGFR activation and expression. Furthermore, cell viability assays showed resistant cells were significantly less sensitive to the anti-EGFR agents when compared to parental cell lines. To further characterise the resistance mechanism in our cells microRNA prediction software identified miR-221 as a negative regulator of EGFR expression. miR-221 was up-regulated in our resistant cell lines, and this up-regulation led to a significant reduction in EGFR expression in both our cultured cell lines and a large cohort of glioblastoma patient tumor tissue.

## Introduction

Glioblastoma (WHO grade 4 astrocytoma) is the most aggressive and lethal primary brain tumor due to its highly invasive and neurologically destructive characteristics^1–3^. Tumor de-bulking is vital although the highly invasive nature of glioblastoma cells entails that microscopic disease is inevitably present, making surgical cure impossible^4^. Nonetheless, maximal safe surgical resection, followed by radiotherapy with concurrent and adjuvant temozolomide (TMZ) is the standard of care for glioblastoma patients^5–8^. Despite aggressive treatment the overall prognosis for glioblastoma patients remains very poor with a median survival of 12 months and a 5-year survival of only 10%^7–10^. The poor patient outcomes are partially driven by intrinsic or acquired tumor resistance to the current therapeutic regimen. Therefore, a greater understanding of the molecular changes involved in tumor resistance to TMZ and irradiation is vital to assist in improving treatment options for glioblastoma patients. There is substantial evidence that receptor tyrosine kinases provide resistance to both irradiation and chemotherapy in the glioblastoma setting^11–13^. Thus many inhibitors targeting these receptors have been trialled in combination with standard therapies, however to date, none have shown a clinical benefit and all have shown additional toxicity^14–17^.

Overexpression of the epidermal growth factor receptor (EGFR) is observed in many tumor types including glioblastoma. Furthermore, anti-EGFR agents including the monoclonal antibodies cetuximab and panitumumab and the tyrosine kinase inhibitors such as gefitinib, erlotinib or afatinib have all successfully become part of the standard clinical management of several tumors. EGFR gene amplification and subsequent EGFR over-expression occurs in approximately 40% of primary glioblastoma^18–20^. In addition, over-expression of the EGFR is frequently associated (63–75%) with rearrangements of the *EGFR* gene, resulting in tumors expressing both wild-type (wt) and mutated EGFR^18,21–24^. The EGFRvIII variant is most common of these EGFR mutations. The EGFRvIII mutation is not expressed in normal tissue^25–28^, but is observed in approximately 50–60% in patients whose tumors show amplification of wt EGFR^2,23,29^. Importantly, the EGFRvIII has been shown to provide clear proliferative and pro-survival advantages to glioblastoma cells. Despite the clear importance of the wt EGFR and EGFRvIII to glioblastoma progression, and a potential role for the EGFR in providing resistance to radiotherapy and chemotherapy, treatment with cetuximab, gefitinib, erlotinib or afatinib have all largely failed^30–36^. However, many of these trials were performed on patients with recurrent glioblastoma that may express differential receptor tyrosine kinase profiles to the original primary tumor. A recent study evaluated glioblastoma patient samples pre and post treatment with either TMZ or Rindopepimut, a vaccine that consists of an EGFRvIII peptide conjugated to keyhole limpet hemocyanin (KLH), in combination with TMZ. Interestingly, about 60% of post-treatment glioblastoma patient samples displayed reduced EGFRvIII expression compared to their pre-treatment matched tumors^37^.

Here we explore whether EGFR expression is varied in matched treatment-sensitive and resistant glioblastoma cell lines. We demonstrate that sub-populations of TMZ and irradiation resistant glioblastoma cells display reduced EGFR expression compared to their sensitive counterparts. We also show that cells with reduced EGFR expression display greater resistance to TMZ and irradiation compared to matched cells with higher EGFR expression. Lastly, we found that miR-221 is potentially linked with the observed lack of EGFR expression in treatment-resistant glioblastoma cells and is may be a key regulator in glioblastoma resistance.

## Materials and methods

### Antibodies and reagents

The rabbit polyclonal antibody directed against pEGFR, EGFR and GAPDH were all obtained from from Cell Signalling Technology (Danvers, MA). All 4 anti-EGFR inhibitors: Erlotinib, Gefitinib, Afatinib and Lapatinib were purchased from Selleck Chemicals (Houston, TX). TMZ was purchased from Sigma and irradiation was performed at the Walter & Eliza Hall Institute for Medical Research.

### Cell culture and inhibitors

The glioblastoma cell lines U87MG, U251MG, U118MG were purchased from ATCC. The primary glioblastoma cell lines: #20, #28, #35 and #41 were originally derived from 4 patients with histo-pathologically confirmed glioblastoma at the Royal Melbourne Hospital and subsequently modified from neurosphere non-adherent cells to adherent cells grown in monolayer. Use of these cell lines in the laboratory was approved by the Melbourne Health Human Research and Ethics Committee (HREC 2012.219). All cells were maintained as previously described^38^ in Dulbecco’s Modified Eagle’s Medium (Life Technologies; Carlsbad, CA) contained 5% foetal bovine serum (FBS) (Life Technologies), 100 U/ml penicillin and 100 µg/ml streptomycin (Life Technologies). Cells were incubated in a humidified atmosphere of 90% air and 10% CO_2_ at 37 °C.

### Generation of resistant cells

U251MG, U87MG and U118MG cells were co-cultured with continuous, increasing doses of TMZ for > 4 months until treatment selected populations of cells (designated U251R, U87R and U118R) displayed resistant to concentrations of 1 mM. Specifically, cells were cultured in an initial dose of 0.1 mM TMZ with fresh medi containing TMZ added weekly. This dose of TMZ was increased to 0.2 mM then 0.5 mM then finally 1 mM over the course of the 4 month treatment. Using a similar protocol as above, #41 cells were co-cultured with continuous, increasing doses of TMZ for > 4 months and simultaneously treated with radiation (5 Gy) monthly until treatment selected populations of cells (designated #41R) displayed resistance to the combination treatment of TMZ and irradiation.Cell viability assays were performed to analyse if cells were resistant after the 4 month co-culture protocol.

### Generation of cells with varying levels of EGFR

U87MG and U251MG cells were seeded at an initial concentration of 1 × 10^3^ cells/ml and serially diluted 1:2 across a 96-well plate with DMEM to isolate wells containing single cells only. These single cell clones were then allowed to proliferate, and expanded populations were scaled up until they were cultured in T75 flasks. Analysis of cell populations from single cell origin for EGFR expression was performed by western blot and qPCR. U87MG cells with reduced EGFR expression compared to parental were designated U87-SCD while U251MG cells with increased EGFR expression compared to parental were designed U251-SCD.

### Cell transfection with miR-221 mimic

The miR-221 mimic (cat no.: 4464066) and the miRNA mimic negative control (cat no.: 4464058) were purchased from Ambion, Austin, TX and prepared according to manufacturer’s protocol. A 3:1 ratio of FuGENE (Promega, Madison, WI): miRNA mimic was generated so that the final concentration of mimic was 25 nM. This mixture was combined with 3 × 10^4^/well and cells were allowed to adhere overnight.

### Cell viability assays

Cells were plated in 96-well plates and allowed to adhere overnight. Triplicate wells were treated with varying concentrations of TMZ, irradiation and/or inhibitors where indicated for 0–7 days. Cells were then lysed and cell viability relative to an appropriate control was determined using a commercially available Cell Titer-Glo kit (Promega) following manufacturer’s instructions and as previously described^39^. Cell lysates were read on a bioluminometer.

### Western blotting

Cells were lysed in a lysis buffer (50 mM Tris (pH 7.4), 150 mM NaCl, 1% Triton-X-100, 50 mM NaF, 2 mM MgCl_2_, 1 mM Na_3_VO_4_ and protease inhibitor cocktail (Roche; Basel, Switzerland)) and clarified by centrifugation (13,000 *g* for 15 min at 4 °C). Proteins were then separated by SDS-PAGE (Life Technologies), blotted onto nitrocellulose and probed with the indicated primary antibodies. The signal was visualized using an ECL chemiluminescence detection kit (GE Healthcare; Chicago, IL) following incubation with appropriate secondary antibodies (Biorad Laboratories; Hercules, CA) as previously described^39^.

### RNA extraction and RT-PCR

Cells were seeded in 6-well plates and allowed to adhere overnight. Following cell treatments and/or transfections, total RNA was extracted with the RNeasy Mini Kit (Qiagen; Hilden, Germany) following the manufacturer’s instructions. Similarly, RNA was first extracted from Glioblastoma samples on formalin-fixed, paraffin-embedded (FFPE) slides by using PureLink FFPE Total RNA Extraction Kit (Invitrogen, cat# KI560-02) and the manufacturer’s instructions were followed, including performing the DNA digestion step prior to reverse transcription. Reverse transcription was performed using the High Capacity RNA-to-cDNA Kit (Applied Biosystems; Waltham, MA) or Taqman microRNA reverse transcription kit (Cat # 4366596) following the protocol provided from the manufacturer. Reverse Transcription-PCR was performed using the GeneAmp PCR System 2400 (Perkin Elmer, Waltham, MA) under the conditions of 37 °C for 60 min and 95 °C for 5 min at a reaction volume of 20 µL. In order to quantify the transcripts of the genes of interest, real-time PCR was performed using the ViiA 7 Real-Time PCR system (Applied Biosystems) for EGFR (Applied Biosystems, Hs01076090_m1) and GAPDH (Applied Biosystems, Hs02758991_g1). Amplified RNA samples was calculated using the 2^−∆∆CT^ method^40^.

### Phospho-RTK array

Cells were seeded at 1 × 10^6^ cells per well in a 10 cm dish for both parental and resistant cell lines. Cells were allowed to adhere overnight. The plate designated to be treated was then irradiated with 5 Gy and incubated for approximately 30 min after which the media was removed and cells were exposed to fresh media containing the appropriate concentration of TMZ. The treated plate was then incubated for 7 days. Cells were lysed and protein concentration was quantified using the Pierce BCA Protein Assay Kit (Thermo Fisher Scientific). A concentration of 300ug was used to prepare the cell lysates and the phospho-RTK levels were analysed using the Phospho-Receptor Tyrosine Kinase Array Kit (R&D Systems, Minneapolis, MN) according to the manufacturer’s instructions. Signal intensity was detected and measured using the ECL chemiluminescence detection kit and analysed by comparing the pixel density on the developed film with the coordinates of the array template.

### OncoLnc (TCGA) and patient samples

TCGA gene expression data was obtained from using the OncoLnc database (www.oncolnc.org). For a given gene, the gene ID was entered and ‘GBM’ was selected. Patients belonging to either the lower and upper 25th percentiles were chosen for the analysis. FFPE sections were received from archived glioblastoma confirmed tumor tissue from 105 primary glioblastoma patients removed during surgery at the Royal Melbourne Hospital (RMH) or Melbourne Private Hospital (MPH). To the best of our knowledge, patients did not receive treatment (TMZ and/or RT) prior to surgical resection of the tumours analysed in this study. The use of this tissue was approved by the Human Research Ethics Committee from Melbourne Health (HREC 2012.136 and HREC 2009.114). Survival data were obtained from the prospectively collected RMH/MPH Central Nervous System (CNS) Tumor Database, part of the Australian Comprehensive Cancer Outcomes Research Database (ACCORD).

### MicroRNA target prediction

Two microRNA target prediction programs were used: TargetScan (www.targetscan.org) and microRNA.org. For the TargetScan analysis the human species was selected before submitting the mRNA transcript of interest. All other parameters were default.

### Statistical analysis

The statistical analyses for all western blots, qRT-PCR and cell viability assays was conducted with an unpaired, two-tail Student’s t-test for significance and a minimum threshold of *p* < 0.05 was chosen to determine significance. The survival analyses from OncoLnc used a log-rank t-test to determine significance and data was displayed on a Kaplan–Meier plot.

## Results

### Treatment resistant cells display reduced EGFR

**Figure 1:**
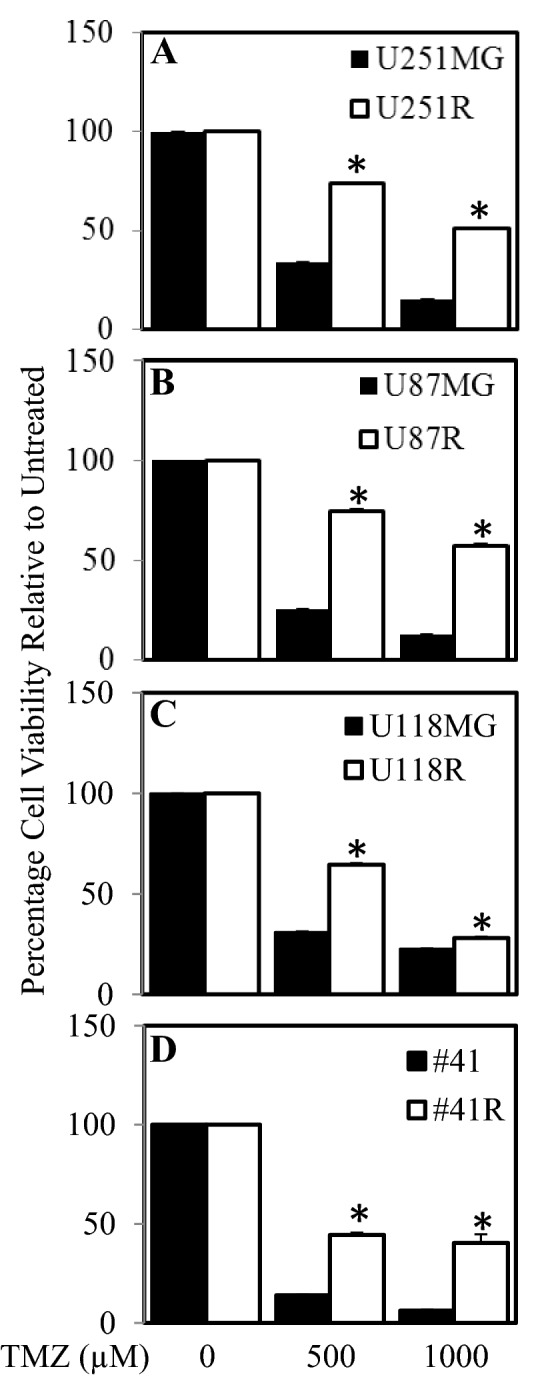
Generation of glioblastoma cells with increased resistance to TMZ and irradiation. (**A**) U251MG, (**B**) U87MG, (**C**) U118MG, (**D**) #41 cells were treated with continuous, increasing doses of TMZ and in the case of #41 TMZ and irradiation for up to 4 months and then assessed for cell viability in comparison to control passage matched parental cells. Parental (■) and Resistant cells (□), were treated with 0, 500 and 1000 µM TMZ for 72 h. Cell viability was then determined using a commercially available Cell Titer-Glo kit and samples read on a bioluminometer. Data is expressed as % viability compared to untreated control cells ± S.D.

**Figure 2:**
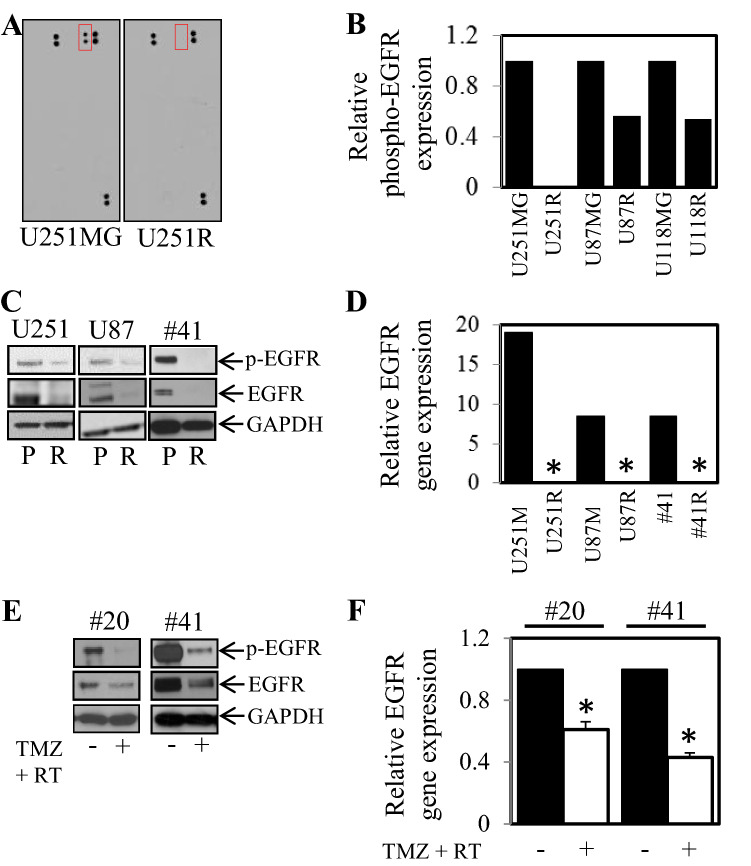
TMZ and irradiation treatment leads to a reduction in EGFR phosphorylation and expression in surviving cells. Cells with acquired resistance to temozolomide were assessed for active receptor tyrosine kinases using an RTK antibody arrays. Reduction in phospho-EGFR is shown in the red box. (**A**) U251R compared to U251MG cells (marked with a red box) and (**B**) graphically in U251R, U87R and U118R compared to their sensitive parental counterparts. (**C**) Parental and Resistant cells were probed for phospho and total levels of EGFR and GADPH expression by western blot and (**D**) assessed for EGFR gene expression by qPCR; *p < 0.05 relative to control. #20 and #41 cell were treated with TMZ (1 mM) and irradiation (5 Gy) for 7 days and then **E.** probed for phospho and total levels of EGFR and GADPH expression by western blot and (**F**) assessed for EGFR gene expression by qPCR; *p < 0.05 relative to control.

Four human glioblastoma cell lines (U251MG, U87MG, U118MG, #41) were treated with increasing doses of TMZ (and in the case of #41; TMZ and irradiation). Following 4 months of treatment cells displayed enhanced resistance to TMZ compared to matched cultured cells that were not treated. These treatment-resistant cells were designated R—for Resistant treatment selected population of cells (Fig. 1). Following the successful generation of resistant cells, we next determined if there were detectable changes in phosphorylation status of a large series of cancer related receptor tyrosine kinases between the sensitive and resistant cell lines. To examine possible changes in receptor tyrosine kinase activity we employed the RTK kinase array comparing resistant and sensitive counterparts. Changes in EGFR phosphorylation status was the most consistent and striking in our panel of RTKs tested. Therefore we focused on changes in EGFR phosphorylation and expression. U251MG, U87MG and U118MG resistant cells (U251R, U87R and U118R) showed less phosphorylated EGFR (pEGFR) levels compared to the matching sensitive parental cell lines (Fig. 2A,B). Verification of these results by western blotting demonstrated that both phosphorylated and total EGFR expression was reduced in U251R, U87R and #41R cells compared to their parental counterparts (Fig. 2C). In addition, EGFR gene expression was undetectable (below threshold levels) in the U251R, U87R and #41R cells while the 3 sensitive parental counterparts displayed detectable levels of EGFR gene expression (Fig. 2D).

As we observed reduced EGFR expression and activity in cells that had been treated long-term with TMZ and irradiation, we next examined RTK phosphorylation status after short-term exposure (0–7 days of treatment). #20 cells treated with TMZ and irradiation caused reduced RTK phosphorylation across many RTK’s tested (Supplementary Fig. 1). Consistently, reduced phosphorylation and total expression of EGFR (Fig. 2E) as well as reduced EGFR gene expression (Fig. 2F) was observed after short-term exposure to TMZ and irradiation in primary cell lines #20 and #41 compared to untreated cells. The reduction of EGFR by the short-term treatment (0–7 days) of TMZ and irradiation was both dose and time dependent (Supplementary Fig. 2).

### Treatment resistant cells are less sensitive to EGFR inhibitors

**Figure 3:**
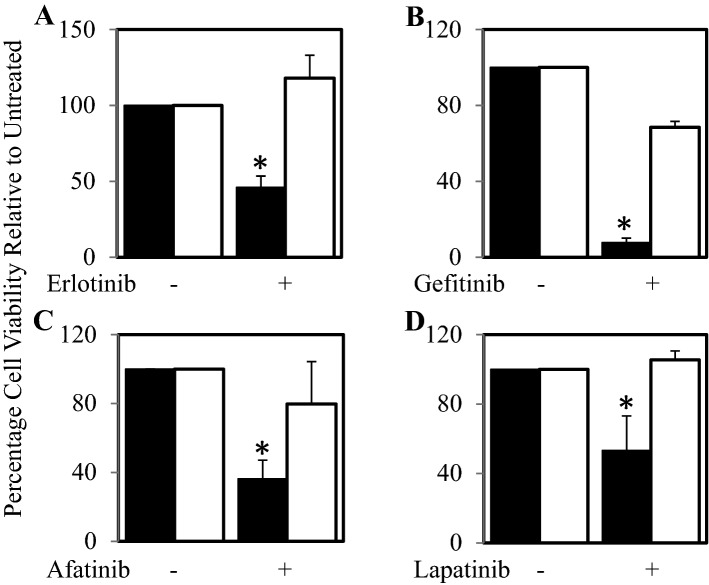
#41R cells are more resistant to anti-EGFR inhibitors compared to control #41 cells. #41 (■) and #41R cells (□) were treated with ± 1 µM. (**A**) Erlotinib, (**B**) Gefitinib, (**C**) Afatinib or (**D**) Lapatinib for 72 h. Cell viability was then determined using a commercially available Cell Titer-Glo kit and samples read on a bioluminometer. Data is expressed as % viability compared to untreated control cells ± S.D.

Given that treatment resistant cells had reduced EGFR expression levels compared to parental cell lines we hypothesised that resistant cells will be less sensitive to EGFR inhibitors. Both parental and #41R cells were exposed to 1 µM of erlotinib, gefitinib, afatinib and lapatinib for 72 h, four FDA approved agents that can inhibit EGFR and have been tested in glioblastoma clinical trials. Cell viability assays confirmed our hypothesis and showed that the parental cells were significantly more sensitive to erlotinib (Fig. 3A), gefitinib (Fig. 3B), afatinib (Fig. 3C) and lapatinib (Fig. 3D) than their resistant counterparts.

### Pre-existing populations of cells with varied levels of EGFR expression display differing sensitivity to TMZ and RT

**Figure 4:**
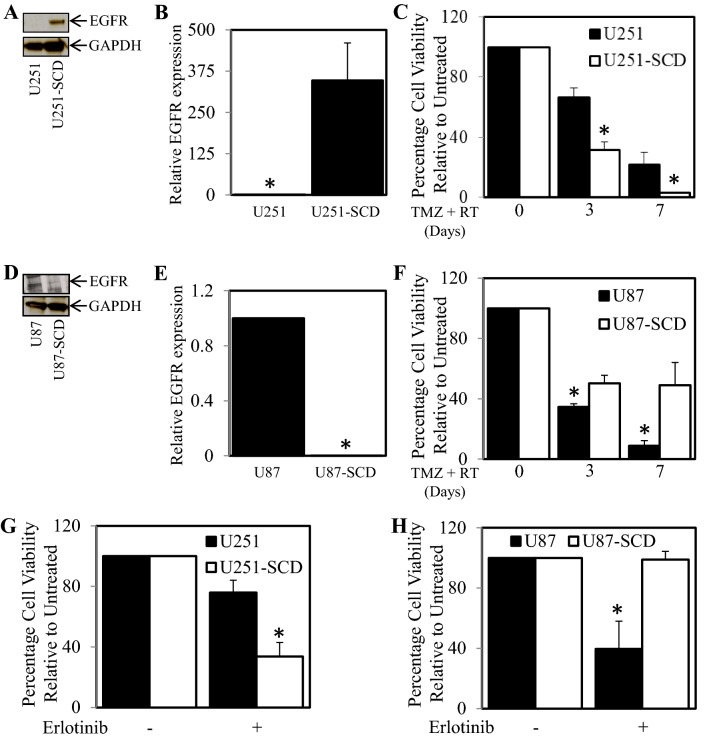
Sub-populations of cells originally Isolated from Single cell dilution display varying sensitivity to TMZ and irradiation based on their EGFR expression. U251MG and U251-SCD cells were (**A**) probed for EGFR and GADPH expression by western blot and (**B**) assessed for EGFR gene expression by qPCR; *p < 0.05 relative to control. (**C**) U251MG (■) and U251-SCD cells (□) were treated with TMZ (1 mM) and irradiation (5 Gy) for 0, 3 and 7 days. Cell viability was then determined using a commercially available Cell Titer-Glo kit and samples read on a bioluminometer. Data is expressed as % viability compared to untreated control cells ± S.D. U87MG and U87-SCD cells were (**D**) probed for EGFR and GADPH expression by western blot and **E.** assessed for EGFR gene expression by qPCR; *p < 0.05 relative to control. **F.** U87MG (■) and U87-SCD cells (□) were treated with TMZ (1 mM) and irradiation (5 Gy) for 0, 3 and 7 days. Cell viability was then determined using a commercially available Cell Titer-Glo kit and samples read on a bioluminometer. Data is expressed as % viability compared to untreated control cells ± S.D. **G.** U251MG (■) and U251-SCD cells (□) and **H.** U87MG (■) and U87-SCD cells (□) were treated ± 1 µM Erlotinib for 72 h. Cell viability was then determined using a commercially available Cell Titer-Glo kit and samples read on a bioluminometer. Data is expressed as % viability compared to untreated control cells ± S.D.

We have found that treatment of TMZ and irradiation led to the generation of a treatment selected population of cells that displayed reduced EGFR expression and greater resistance to TMZ (1 mM) and RT (5 Gy) compared to the parental cell population. However, we could not conclude as to whether the TMZ and RT treatment was selecting pre-existing populations of low expressing EGFR cells or whether the treatment was causing cells to reduce EGFR expression as an adaptive measure. Therefore we next investigated whether we could isolate and then populate single cell colonies with increased or decreased EGFR expression to determine whether they display differential treatment sensitivity compared to the overall population. Following, serial cell dilution to cultivate colonies originating from a single cell and a period of 3 months to allow single cells to re-populate we were successfully able to obtain a single cell derived population of U251 (designated U251-SCD; for single cell derived) and U87 cells (designated U87-SCD). When comparing the total EGFR expression of parental U251 with the U251-SCD we observed higher EGFR protein and gene expression levels in the U251-SCD cells (Fig. 4A,B). Consistent with our earlier findings, cell viability assays showed that the U251-SCD cells were significantly more sensitive to a treatment combination containing 5 Gy irradiation and 1 mM TMZ (Fig. 4C) after 3 and 7 days of treatment. Conversely, U87-SCD displayed lower EGFR protein and gene expression (Fig. 4D,E) compared to the parental U87 cells. Consistently, the U87-SCD cell line with lower EGFR expression was significantly more resistant to 5 Gy irradiation and 1000 µM TMZ compared to the parental population (Fig. 4F). Consistently, Erlotinib was more effective against U251-SCD (with higher EGFR expression) compared to control U251 cells (Fig. 4G), while erlotinib was less effective against U87-SCD (with lower EGFR) compared to control U87 cells (Fig. 4H).

### MiR-221 is over-expressed in treatment resistant cells

**Figure 5:**
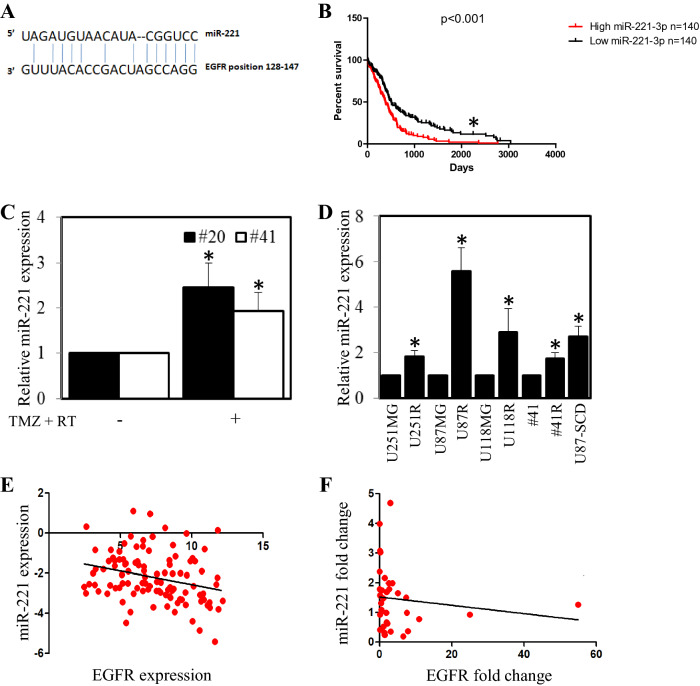
Micro-RNA-221 is inversely correlated with EGFR expression in Glioblastoma cell lines and patient samples. (**A**) Base pairing between miR-221 target sequence and potential binding site with EGFR (sourced from microRNA.org). (**B**) The relationship between high (red dashed line) and low (green dashed line) miR-221 expression with patient survival was determined through mining a Oncolnc TCGA dataset. Kaplan–Meier survival curves were evaluated from the TCGA, n = 140; *p < 0.05. (**C**) #20 and #41 cell were treated with temozolomide and irradiation for 7 days and then assessed for miR-221 expression by qPCR; *p < 0.05 relative to control. (**D**) Treatment-sensitive and resistant matched cells were assessed for miR-221 expression by qPCR; *p < 0.05 relative to control. (**E**) EGFR and miR-221 expression in 105 primary tumors were assessed and dCT values were plotted. (**F**) The fold-change of miR-221 and EGFR levels at recurrence of 34 patients compared to paired primary tumors.

As we observed that the EGFR expression levels were reduced in treatment resistant cells, we next aimed to determine whether this may be due to changes in the expression of miRNA that regulate EGFR expression. To achieve this aim we initially examined two miRNA bioinformatics databases (miRSVR and TargetScan) to identify miRNA that are predicted to target and negatively regulate EGFR expression. The miRNA bioinformatics databases yielded several potential miRNA candidates. However, we chose to further examine the role of miR-221 as it displayed one of the highest probabilities of preferentially conserved targeting of EGFR and its expression significantly correlated with poor survival in Glioblastoma patients (Fig. 5A,B).

We next explored the levels of miR-221 in treated and untreated cells by RT-qPCR. Short-term exposure (0–7 days) to TMZ and irradiation led to an increase in miR-221 levels in both the #20 and #41 primary glioblastoma cells compared to untreated cells (Fig. 5C). In addition long-term resistant cell lines U251R, U87R, U118R and #41R cells (which all had reduced EGFR expression compared to their parental counterparts) and the U87-SCD cell line (which had lower EGFR expression than the U87 parental cell line) had significantly higher miR-221 levels compared to their parental counterparts (Fig. 5D). To determine the expression of miR-221 and EGFR in patients, we assessed the expression of miR-221 and EGFR in a cohort of 105 primary glioblastoma patient samples. A significant inverse correlation between miR-221 and EGFR was observed (Fig. 5E). Patients with a lower miR-221 delta CT (dCT) value (which inversely represents expression) had a higher EGFR dCT value; therefore, an increase in miR-221 expression in primary tumors correlates with a decrease in EGFR expression.

Tumor samples from 34 patients with both primary and recurrent tissue were also assessed. The fold-change levels of miR-221 and EGFR at recurrence, relative to the primary tumor, were plotted on a scatter plot for each matched patient (Fig. 5F). Data analysis revealed a statistically significant inverse correlation between miR-221 fold-change and EGFR fold-change at recurrence. Similarly, an inverse correlation between EGFR and miR-221 expression was observed in four primary cell lines generated from glioblastoma patient tumor tissue (Supplementary Fig. 3).

### MiR-221 over-expression is linked to EGFR down-regulation and increased resistance

**Figure 6:**
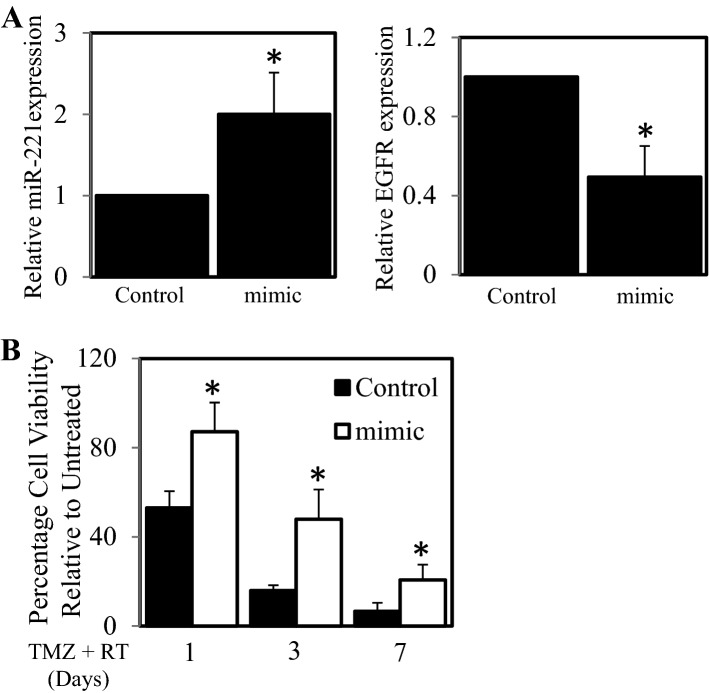
Transfection of miR-221 mimic results in reduced EGFR expression and increased resistance to treatment. The #41 cell line was transfected with control or miR-221 mimic and then assessed for (**A**) miR-221 and EGFR gene expression by qPCR; *p < 0.05 relative to control. (**B**) #41 cells transfected with control (■) or miR-221 mimic (□) were treated with TMZ (1 mM) and irradiation (5 Gy) for 1, 3 and 7 days. Cell viability was then determined using a commercially available Cell Titer-Glo kit and samples read on a bioluminometer. Data is expressed as % viability compared to untreated control cells ± S.D.

Given that our treatment resistant Glioblastoma cells have low EGFR levels and higher miR-221 expression compared to their treatment-sensitive counterparts we next aimed to establish whether miR-221 directly regulated EGFR expression and played a role in the observed treatment resistance. To investigate this we transfected #41 parental cells with miR-221 mimic and found that the miR-221 levels had significantly increased while the level of EGFR gene expression had significantly decreased (Fig. 6A). Importantly, #41 cells transfected with the miR-221 mimic (and thereby had reduced EGFR expression) displayed significantly greater survival when treated with TMZ (1 mM) and RT (5 Gy) compared to un-transfected cells or cells transfected with scrambled control after 1, 3 and 7 days of treatment (Fig. 6B).

## Discussion

Changes to the molecular drivers of cancer occur frequently when challenged with conventional and targeted therapy with the adaptive changes often favouring tumor survival and resistance to treatment. The EGFR has long been recognised as a critical initiator of signal transduction for the development, progression and recurrence of many tumors including glioblastoma^10,41,42^. However, the exact change to the expression levels of EGFR during and post-treatment with TMZ and irradiation in the glioblastoma setting is not well understood.

Our current results indicate that down-regulation of the EGFR gene and protein expression is a feature in our cell lines after standard therapy with TMZ and irradiation and occurred within days. Interestingly, this reduction in EGFR expression was far more evident after TMZ treatment compared to the reduction seen in cells treated with irradiation only. It has been demonstrated that berberine-induced down-regulation of EGFR causes senescence in Glioblastoma cells^43^. This initially suggests that the surviving cells in our experiments might have preferentially shifted from a highly proliferative state to a senescence-like state. However our resistant cell lines proliferated at an equal or greater rate to their treatment-sensitive parental counterparts at least in optimised culture conditions in vitro*.* Similar to our current results, others have reported the reduction of EGFRvIII expression in post-treated glioblastoma patient samples^37,44^ *.*

It should be noted that although we saw a clear reduction in EGFR phosphorylation and expression post TMZ and irradiation treatment and thus the EGFR was the major focus of this article, there may be many other changes to the activity and expression levels of both receptor tyrosine kinase and other key molecules that predict and mediate glioblastoma cell resistance to treatment. It is long been established that the DNA repair protein, O^6^ methylguanine-DNA methyltransferase (MGMT), is a predictive biomarker of response to temozolomide treatment in patients with glioblastoma^45^. Similarly, c-MET^46,47^, IGF-R^48^ and PDGFR^49^ have been identified as potential drivers of resistance to temozolomide and or radiotherapy. Nonetheless, our major focus in this study was the EGFR.

This reduction in EGFR following TMZ and irradiation may assist in providing some explanation as to why targeting the EGFR in clinical trials of recurrent glioblastoma patients have largely failed^30–36^. We speculate that the post-treatment selection of a populations of cells that have low EGFR expression do not require significant EGFR-driven signaling to survive and proliferate, leading to a recurrent cell population that is refractory to anti-EGFR inhibitors. As such agents such as erlotinib, gefitinib, afatinib or lapatinib do not inhibit glioblastoma cells post-TMZ and irradiation treatment as shown with our glioblastoma cell models. This notion is further strengthened by the single cell dilution experiments we performed. Populations of cells expanded from an original single cell in the overall population displayed reduced EGFR expression and inherent resistance to treatment not only to TMZ and irradiation but also to anti-EGFR inhibition (erlotinib). Our results re-emphasise, the heterogeneous nature of glioblastoma and the consequent challenge to targeted therapy. Although our data does not necessarily invalidate the possibility of acquired resistance contributing to recurrence, it supports the inherent resistance model. Nonetheless, we propose that resistance to EGFR inhibition can be attributed to TMZ and radiotherapy down-regulating EGFR levels leading to the recurrent population being inherently resistant to the supplementary EGFR inhibition.

It is well established that there are several subtypes of glioblastoma based on their gene expression and DNA mutation characteristics and treatment responses (pro-neural, neural, mesenchymal and classical)^50,51^. EGFR amplification and mutations are more often found in glioblastomas in the classical sub-type^52,53^. Our current results indicate that treatment of TMZ and irradiation selects for a population of cells that have reduced EGFR. However, whether this selected population displays varied gene expression profiles other than reduced EGFR and differential sub-types was not assessed here.

Despite increased interest in the role miRNA regulation plays in glioblastoma tumorigenesis, specific miRNA regulation of EGFR in has not been studied extensively. MiR-221 is generally considered an oncomir, stimulating proliferation, invasion and tumorigenesis^54,55^. MiR-221 was found to correlate with glioma grade^56^, while data from the TCGA database indicates that miR-221 expression correlates with poorer survival in glioblastoma patients. A relationship between the EGFR and miR-221 has not yet been reported in glioblastoma; however Garofalo et al. concluded that EGFR modulates miR-221 in gefitinib resistant lung cancer cells after EGFR silencing led to down-regulation of miR-221^57^. Our study differs from Garofalo, placing miR-221 up-stream of EGFR rather than being contingent upon it and this is most likely due to differences in cancer type and cell line. To our knowledge, this is the first report of miR-221 modulating EGFR expression in all cancer types. A previous study by Toraih et al., found that miR-221 expression was higher in glioblastoma patient tumor tissue compared to normal brain tissue^58^. They also found that EGFR gene expression was reduced in the same glioblastoma patient tissue compared to normal brain tissue. However, they did not determine whether an inverse correlation existed between miR-221 and EGFR (i.e.: samples with high miR-221 expression had low EGFR expression), nor whether miR-221 regulated EGFR expression. Importantly our study indicates that this inverse relationship extends into glioblastoma patient samples and not just cell lines.

Interestingly, Brognara and colleagues demonstrated that inhibiting miR-221 leads to an increase in TMZ-induced apoptosis in glioblastoma cell lines: U87, U251 and T98G^59^. However, they did not examine whether reduced miR-221 led to enhanced EGFR expression as we have demonstrated. Nonetheless, our results concur with the findings of Brognara et al. demonstrating that exogenous enhanced miR-221 expression (miR-221 mimic) leads to increased survival following TMZ and irradiation treatment (through the reduction of EGFR expression). Furthermore, we show that glioblastoma cells that have survived long-term, undergo repeated TMZ and irradiation treatment or cells that intrinsically express low EGFR and are resistant to treatment all have high levels of miR-221. The mechanism that drives higher miR-221 levels in some cells is unknown, but we can hypothesise that a small subset of cells within the overall population has higher miR-221 and subsequent low EGFR expression specifically for treatment resistance purposes. These tumour cells then re-populate the tumor post-treatment similarly to the hypothesis that cancer stem cells are treatment refractory and allow for re-establishment of challenged tumors. Overall, our novel results establish miR-221 as a key onco-mir driving both therapy resistance and resistance-initiated glioblastoma recurrence through the regulation of EGFR expression. Our data also presents some evidence for the use of a high miR-221 and low EGFR expression signature as a potential biomarker for poorer survival at recurrence in glioblastoma.

In conclusion, we have demonstrated that down-regulation of EGFR activity and expression is induced by TMZ and radiotherapy. We also show that this treatment-resistant population pre-exists with reduced levels of EGFR prior to treatment suggesting that the Stupp protocol may select for a population of resistant cells that survive independently of EGFR signalling, rendering subsequent treatment with EGFR tyrosine kinase inhibitors ineffective. In addition, we demonstrate that miR-221 is up-regulated in this pre-existing population of treatment resistant cells and that miR-221 can mediate resistance to TMZ and radiotherapy by down-regulating EGFR expression, making it a potential candidate for further strategic targeting. Overall, our current data emphasises the importance of adopting both TMZ and irradiation in pre-clinical models to better characterise glioblastoma biology and translate findings to the clinic.

## Supplementary information

Supplementary information.
