# Supplementary material for: Reduced EGFR and increased miR-221 is associated with increased resistance to temozolomide and radiotherapy in glioblastoma
Source: Sci Rep. 2020 Oct 20;10:17768. doi: 10.1038/s41598-020-74746-x (PMC7576591; doi:10.1038/s41598-020-74746-x)
Supplement: Supplementary file 1 — Supplementary information. [file 41598_2020_74746_MOESM1_ESM.pptx]

## Slide 1
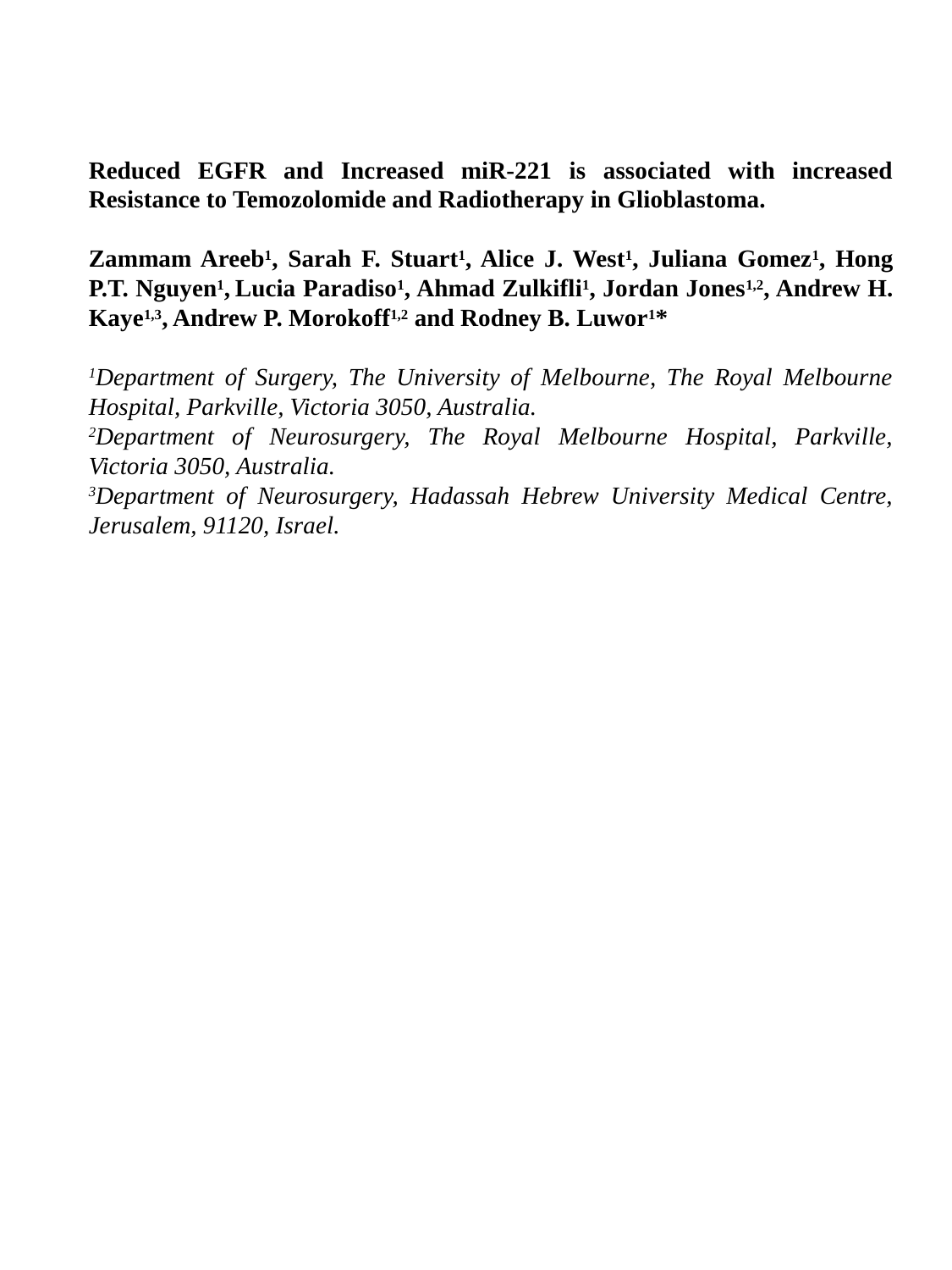

Reduced EGFR and Increased miR-221 is associated with increased Resistance to Temozolomide and Radiotherapy in Glioblastoma.
Zammam Areeb1, Sarah F. Stuart1, Alice J. West1, Juliana Gomez1, Hong P.T. Nguyen1, Lucia Paradiso1, Ahmad Zulkifli1, Jordan Jones1,2, Andrew H. Kaye1,3, Andrew P. Morokoff1,2 and Rodney B. Luwor1*
1Department of Surgery, The University of Melbourne, The Royal Melbourne Hospital, Parkville, Victoria 3050, Australia.
2Department of Neurosurgery, The Royal Melbourne Hospital, Parkville, Victoria 3050, Australia.
3Department of Neurosurgery, Hadassah Hebrew University Medical Centre, Jerusalem, 91120, Israel.

## Slide 2
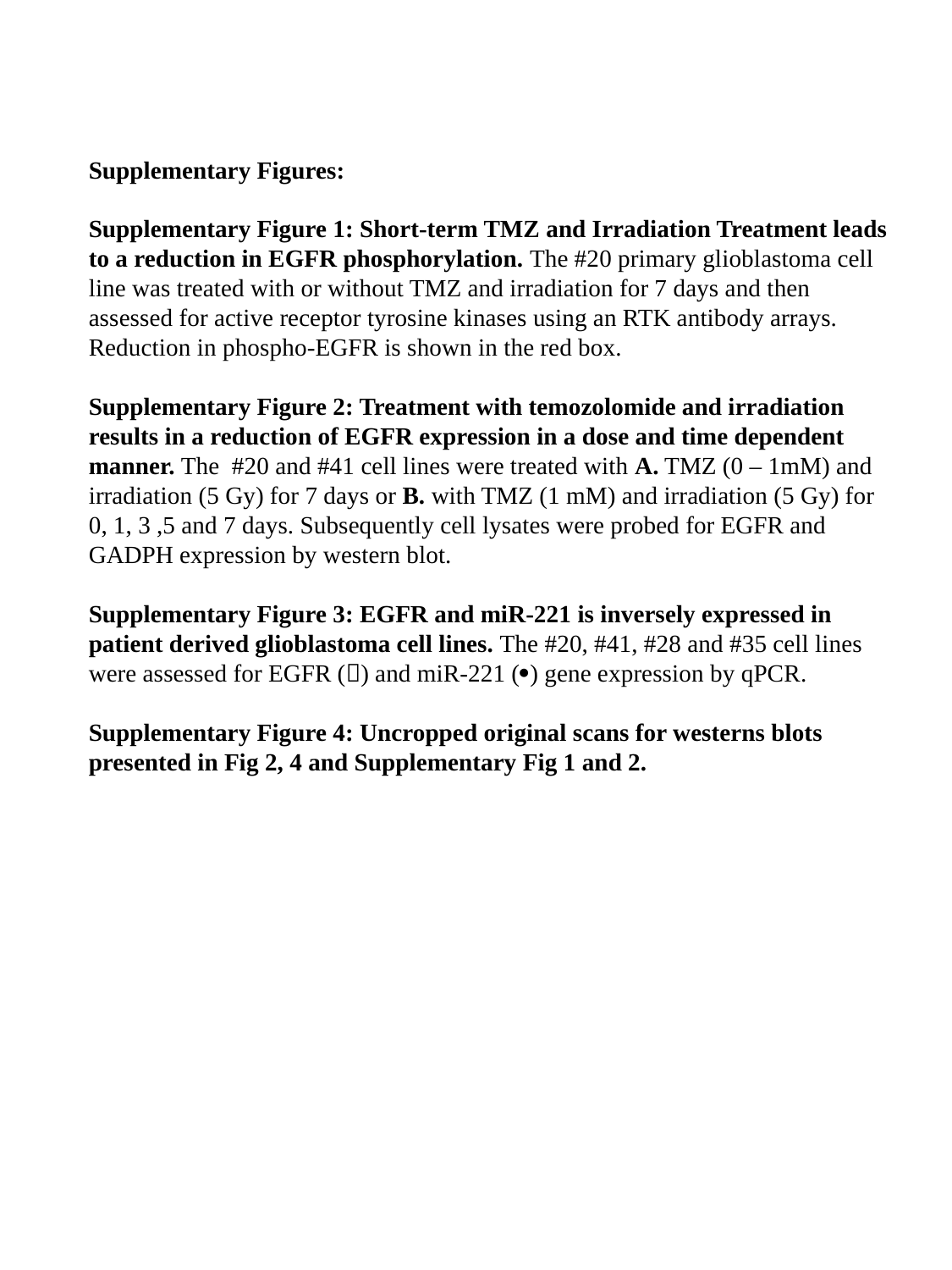

Supplementary Figures:
Supplementary Figure 1: Short-term TMZ and Irradiation Treatment leads to a reduction in EGFR phosphorylation. The #20 primary glioblastoma cell line was treated with or without TMZ and irradiation for 7 days and then assessed for active receptor tyrosine kinases using an RTK antibody arrays. Reduction in phospho-EGFR is shown in the red box.
Supplementary Figure 2: Treatment with temozolomide and irradiation results in a reduction of EGFR expression in a dose and time dependent manner. The #20 and #41 cell lines were treated with A. TMZ (0 – 1mM) and irradiation (5 Gy) for 7 days or B. with TMZ (1 mM) and irradiation (5 Gy) for 0, 1, 3 ,5 and 7 days. Subsequently cell lysates were probed for EGFR and GADPH expression by western blot.
Supplementary Figure 3: EGFR and miR-221 is inversely expressed in patient derived glioblastoma cell lines. The #20, #41, #28 and #35 cell lines were assessed for EGFR () and miR-221 () gene expression by qPCR.
Supplementary Figure 4: Uncropped original scans for westerns blots presented in Fig 2, 4 and Supplementary Fig 1 and 2.

## Slide 3
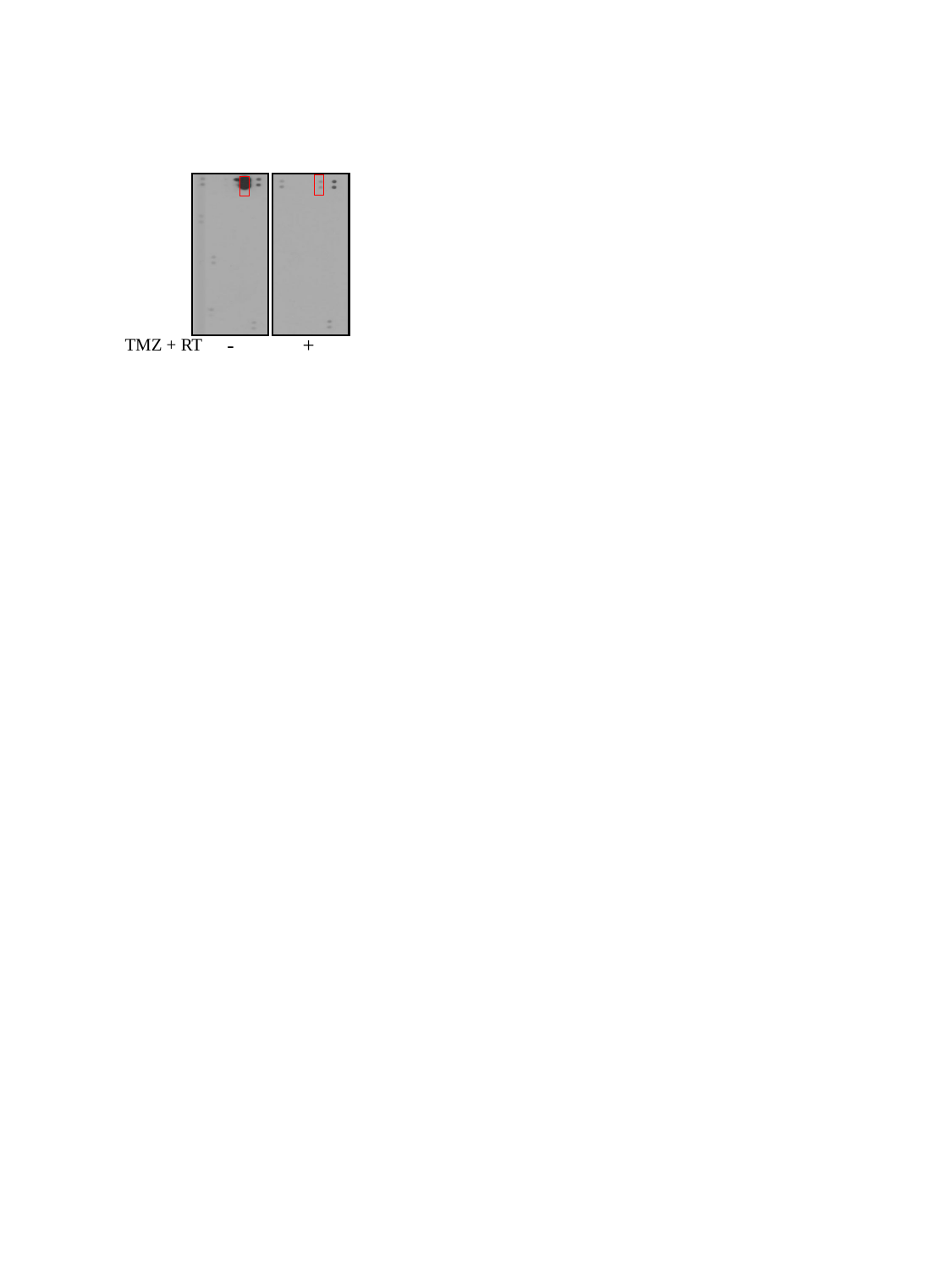

+
-
TMZ + RT

## Slide 4
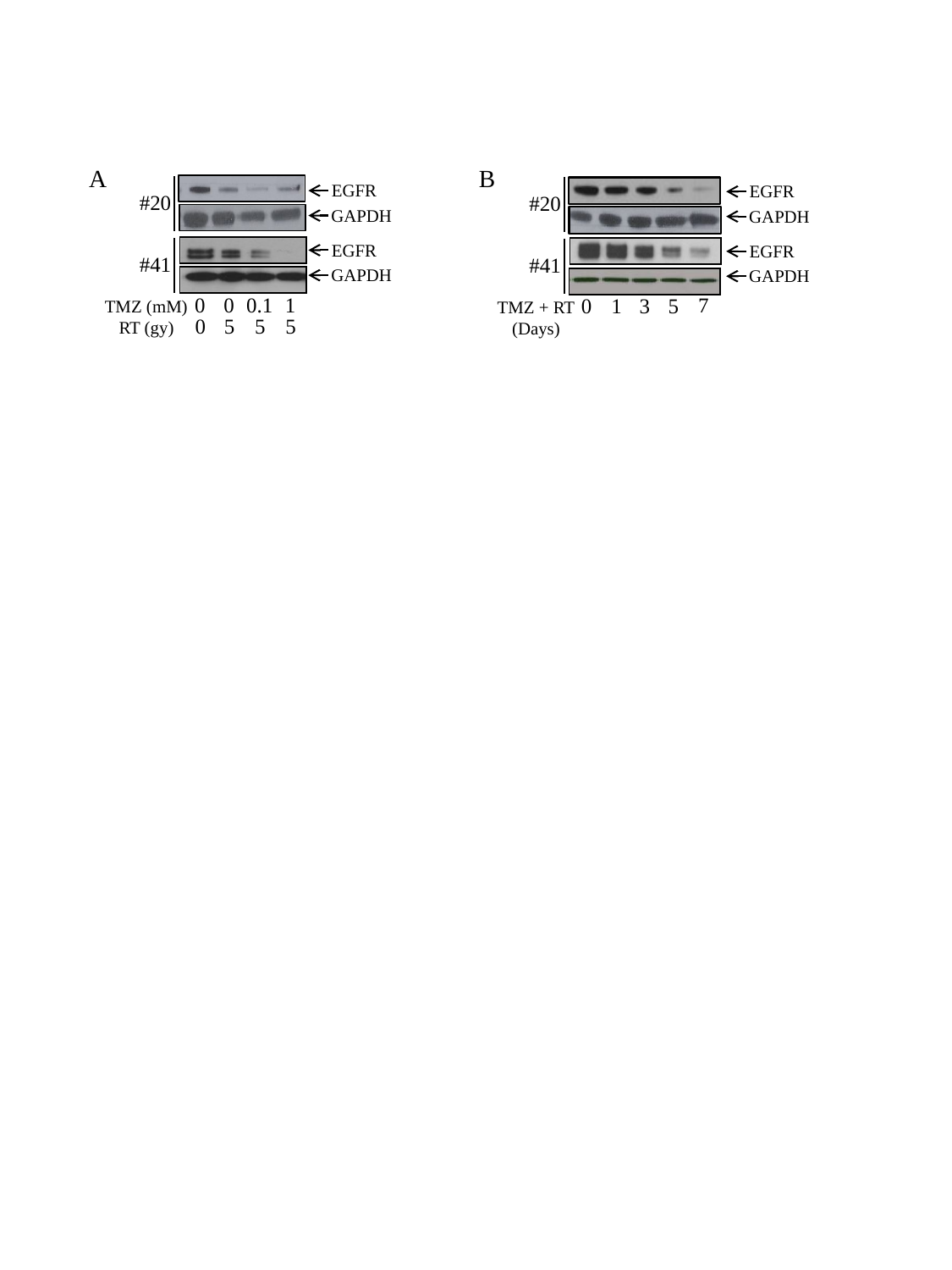

B
EGFR
#20
GAPDH
EGFR
#41
GAPDH
7
0
3
5
1
TMZ + RT
(Days)
A
EGFR
#20
GAPDH
EGFR
#41
GAPDH
0
0.1
1
0
TMZ (mM)
0
5
5
5
RT (gy)

## Slide 5
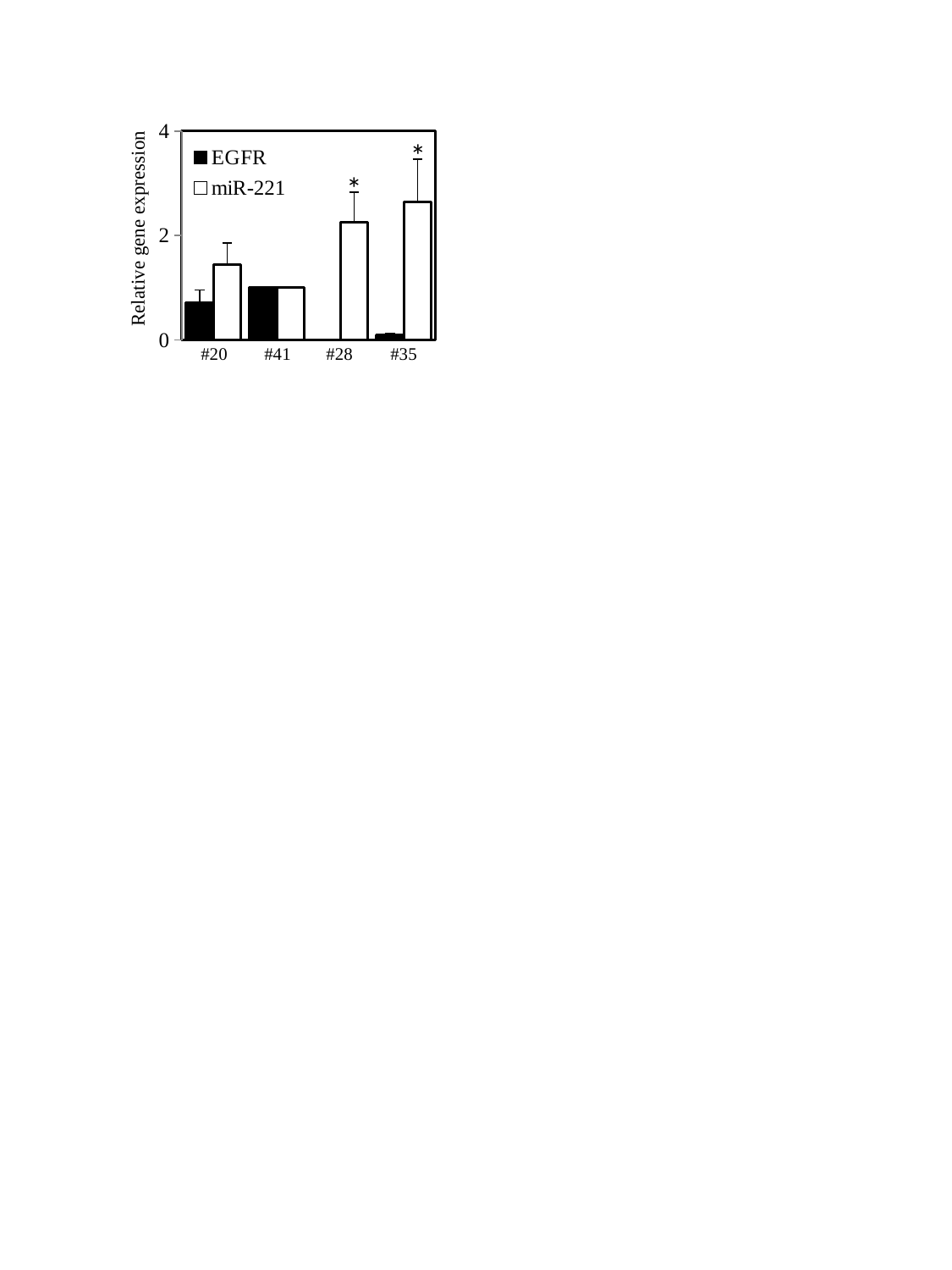

### Chart
| Category | EGFR | miR-221 |
|---|---|---|
| #20 | 0.713333 | 1.440619 |
| #41 | 1.0 | 1.0 |
| #28 | 0.001195 | 2.253334 |
| #35 | 0.089708 | 2.638786 |Relative gene expression
#35
#28
#20
#41
*
*

## Slide 6
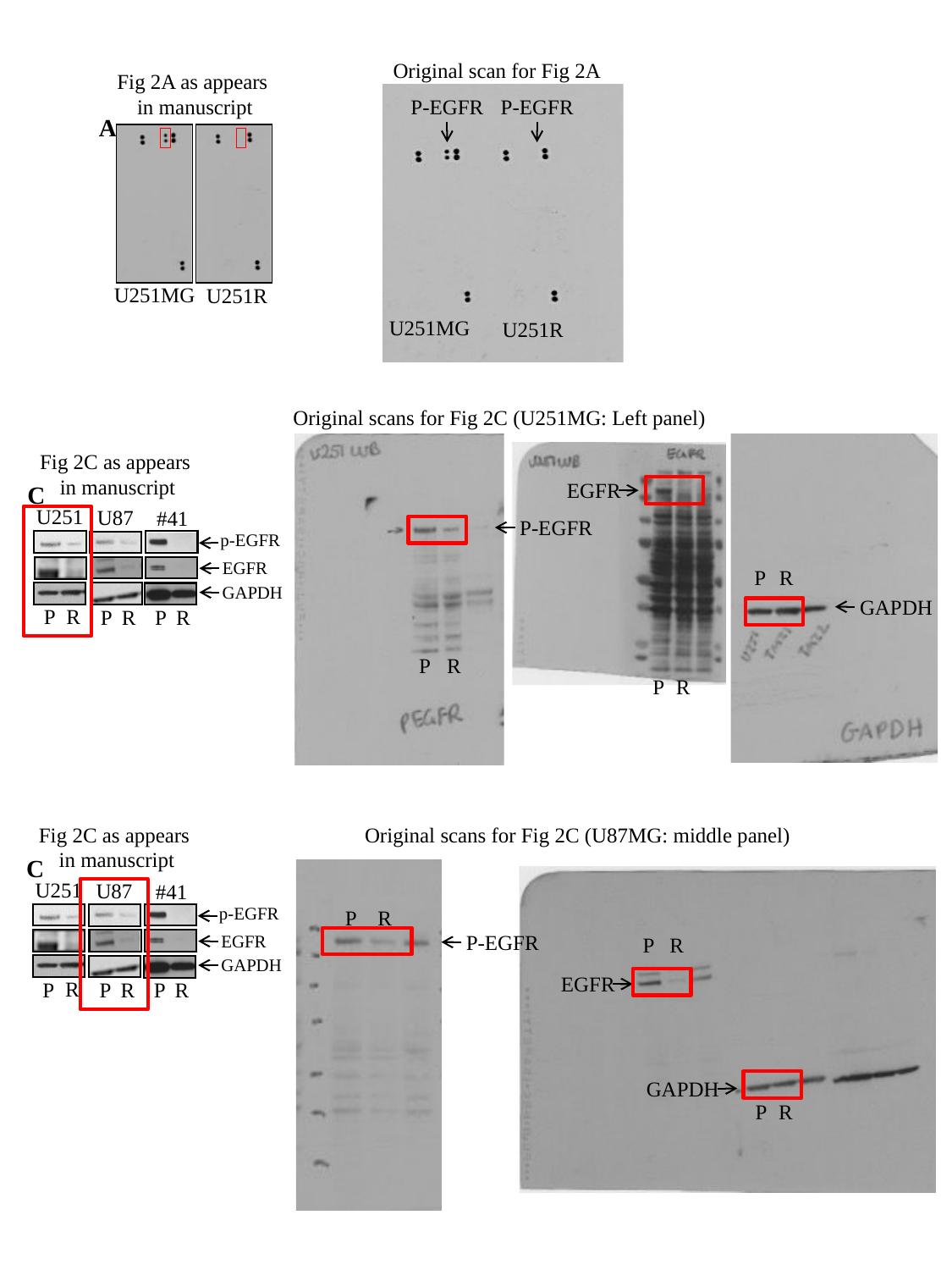

Original scan for Fig 2A
Fig 2A as appears
in manuscript
P-EGFR
P-EGFR
A
U251MG
U251R
U251MG
U251R
Original scans for Fig 2C (U251MG: Left panel)
Fig 2C as appears
in manuscript
EGFR
C
U251
U87
#41
p-EGFR
EGFR
GAPDH
R
P
R
R
P
P
P-EGFR
P
R
GAPDH
P
R
P
R
Fig 2C as appears
in manuscript
Original scans for Fig 2C (U87MG: middle panel)
C
U251
U87
#41
p-EGFR
EGFR
GAPDH
R
P
R
R
P
P
P
R
P-EGFR
P
R
EGFR
GAPDH
P
R

## Slide 7
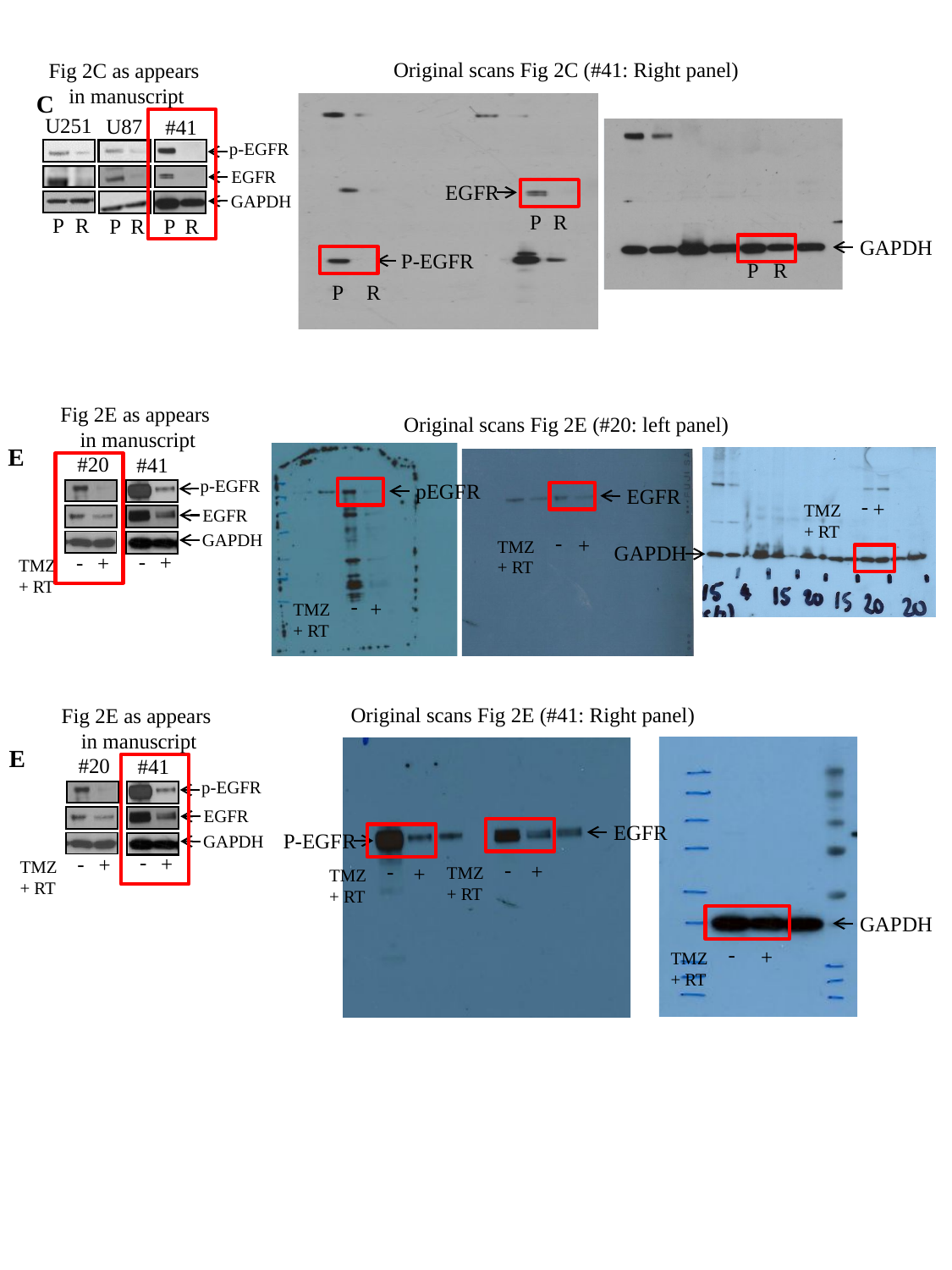

Original scans Fig 2C (#41: Right panel)
Fig 2C as appears
in manuscript
C
U251
U87
#41
p-EGFR
EGFR
GAPDH
R
P
R
R
P
P
EGFR
P
R
GAPDH
P-EGFR
P
R
P
R
Fig 2E as appears
in manuscript
Original scans Fig 2E (#20: left panel)
E
#20
#41
p-EGFR
EGFR
GAPDH
-
-
+
+
TMZ
+ RT
pEGFR
EGFR
-
+
TMZ
+ RT
-
+
TMZ
+ RT
GAPDH
-
+
TMZ
+ RT
Original scans Fig 2E (#41: Right panel)
Fig 2E as appears
in manuscript
E
#20
#41
p-EGFR
EGFR
GAPDH
-
-
+
+
TMZ
+ RT
EGFR
P-EGFR
-
-
+
+
TMZ
+ RT
TMZ
+ RT
GAPDH
-
+
TMZ
+ RT

## Slide 8
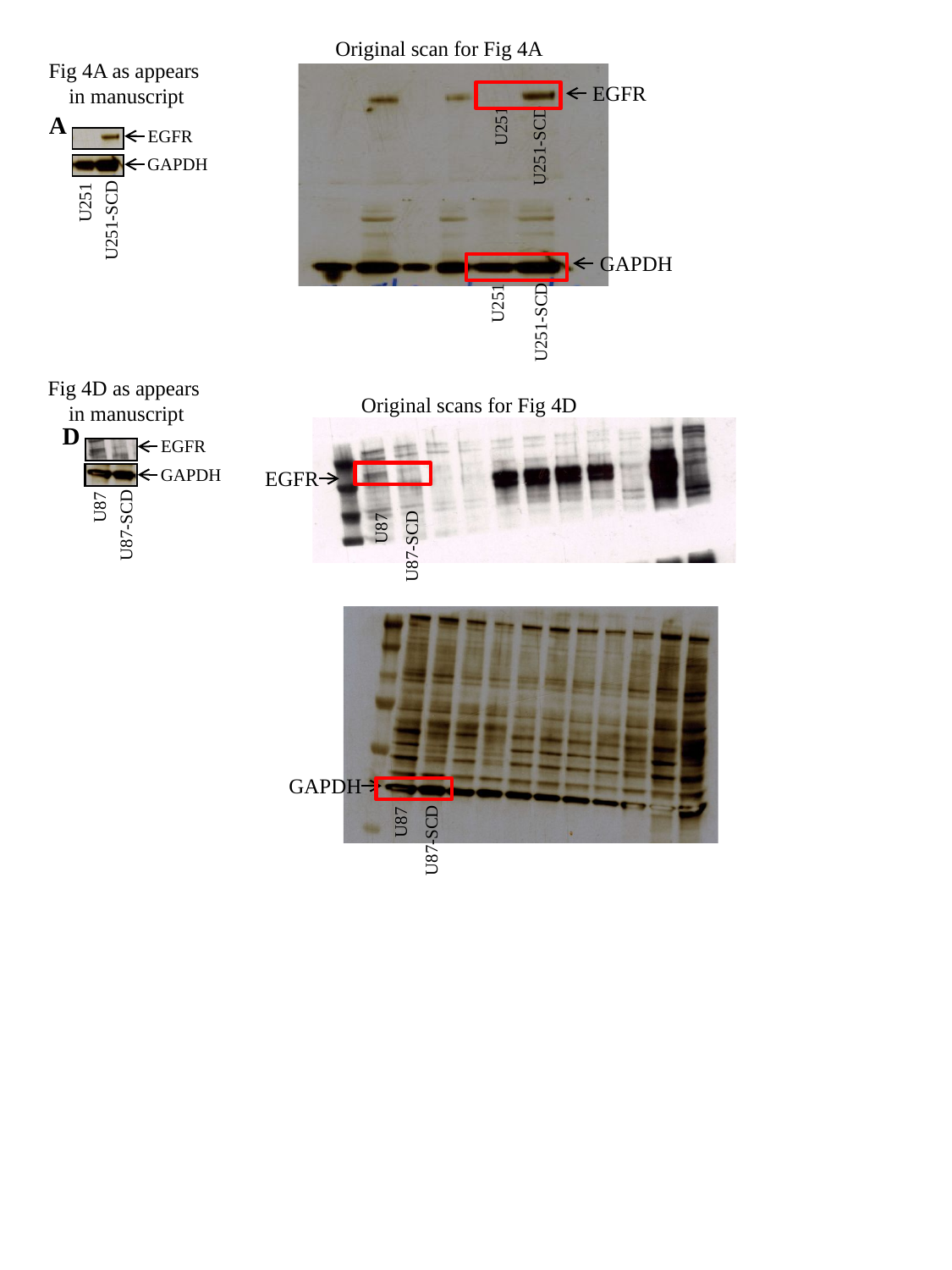

Original scan for Fig 4A
Fig 4A as appears
in manuscript
EGFR
A
EGFR
GAPDH
U251
U251-SCD
U251
U251-SCD
GAPDH
U251
U251-SCD
Fig 4D as appears
in manuscript
Original scans for Fig 4D
D
EGFR
GAPDH
U87
U87-SCD
EGFR
U87
U87-SCD
GAPDH
U87
U87-SCD

## Slide 9
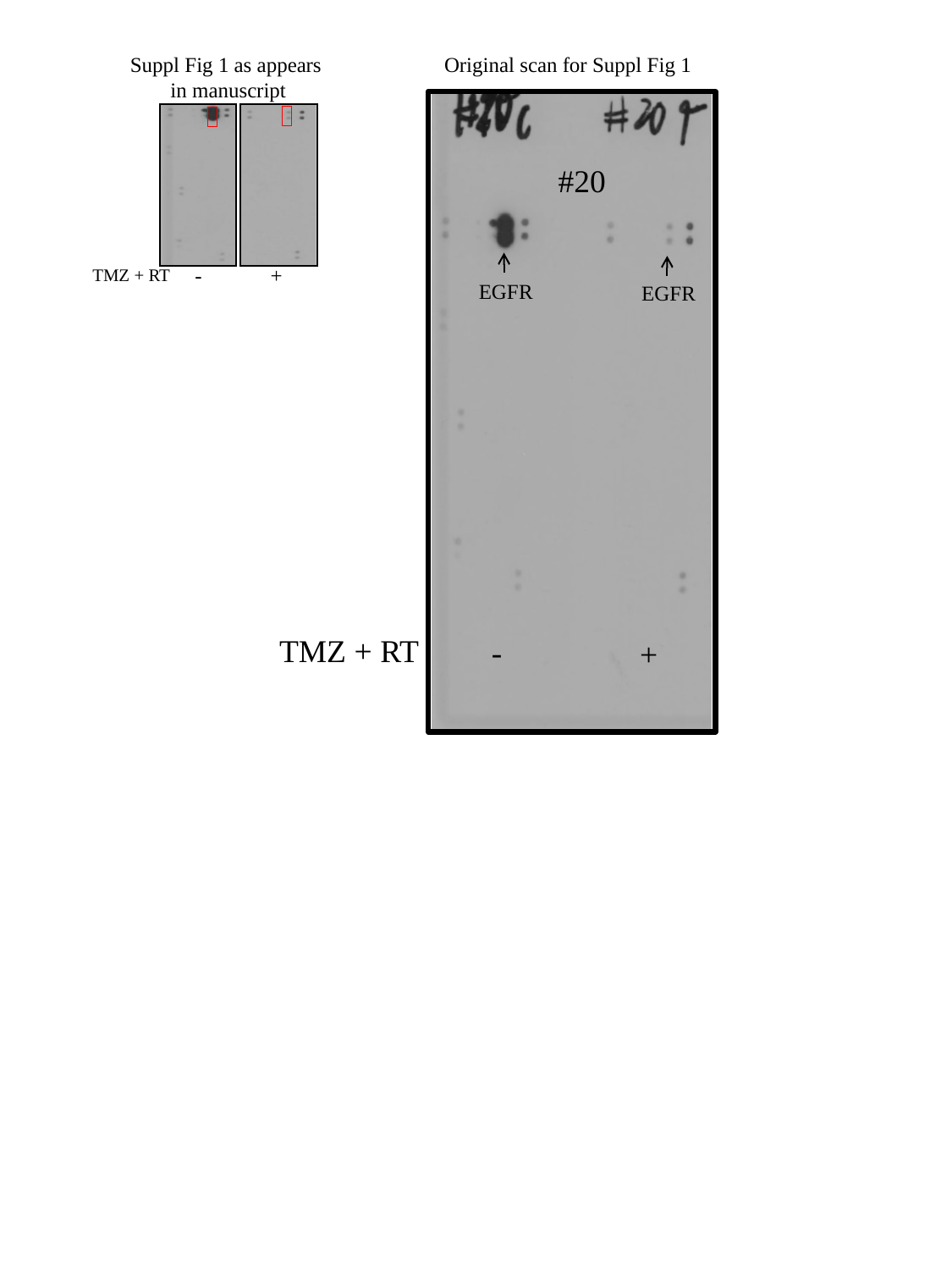

Suppl Fig 1 as appears
in manuscript
Original scan for Suppl Fig 1
+
-
TMZ + RT
#20
EGFR
EGFR
TMZ + RT
-
+

## Slide 10
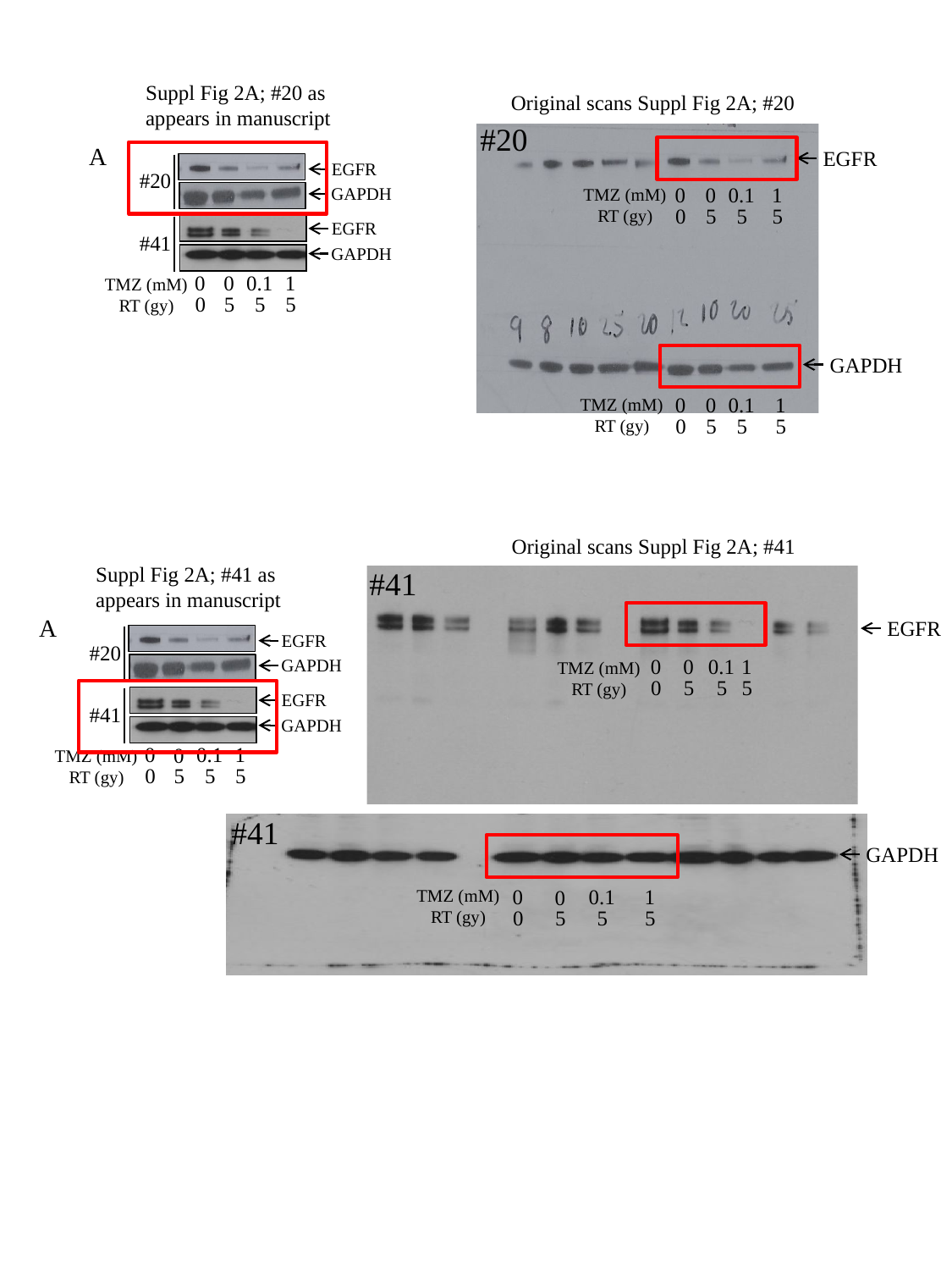

Suppl Fig 2A; #20 as
appears in manuscript
Original scans Suppl Fig 2A; #20
#20
A
EGFR
#20
GAPDH
EGFR
#41
GAPDH
0
0.1
1
0
TMZ (mM)
0
5
5
5
RT (gy)
EGFR
0
0.1
1
0
TMZ (mM)
0
5
5
5
RT (gy)
GAPDH
0
0.1
1
0
TMZ (mM)
0
5
5
5
RT (gy)
Original scans Suppl Fig 2A; #41
Suppl Fig 2A; #41 as
appears in manuscript
#41
A
EGFR
#20
GAPDH
EGFR
#41
GAPDH
0
0.1
1
0
TMZ (mM)
0
5
5
5
RT (gy)
EGFR
0
0.1
1
0
TMZ (mM)
0
5
5
5
RT (gy)
#41
GAPDH
0
0.1
1
0
TMZ (mM)
0
5
5
5
RT (gy)

## Slide 11
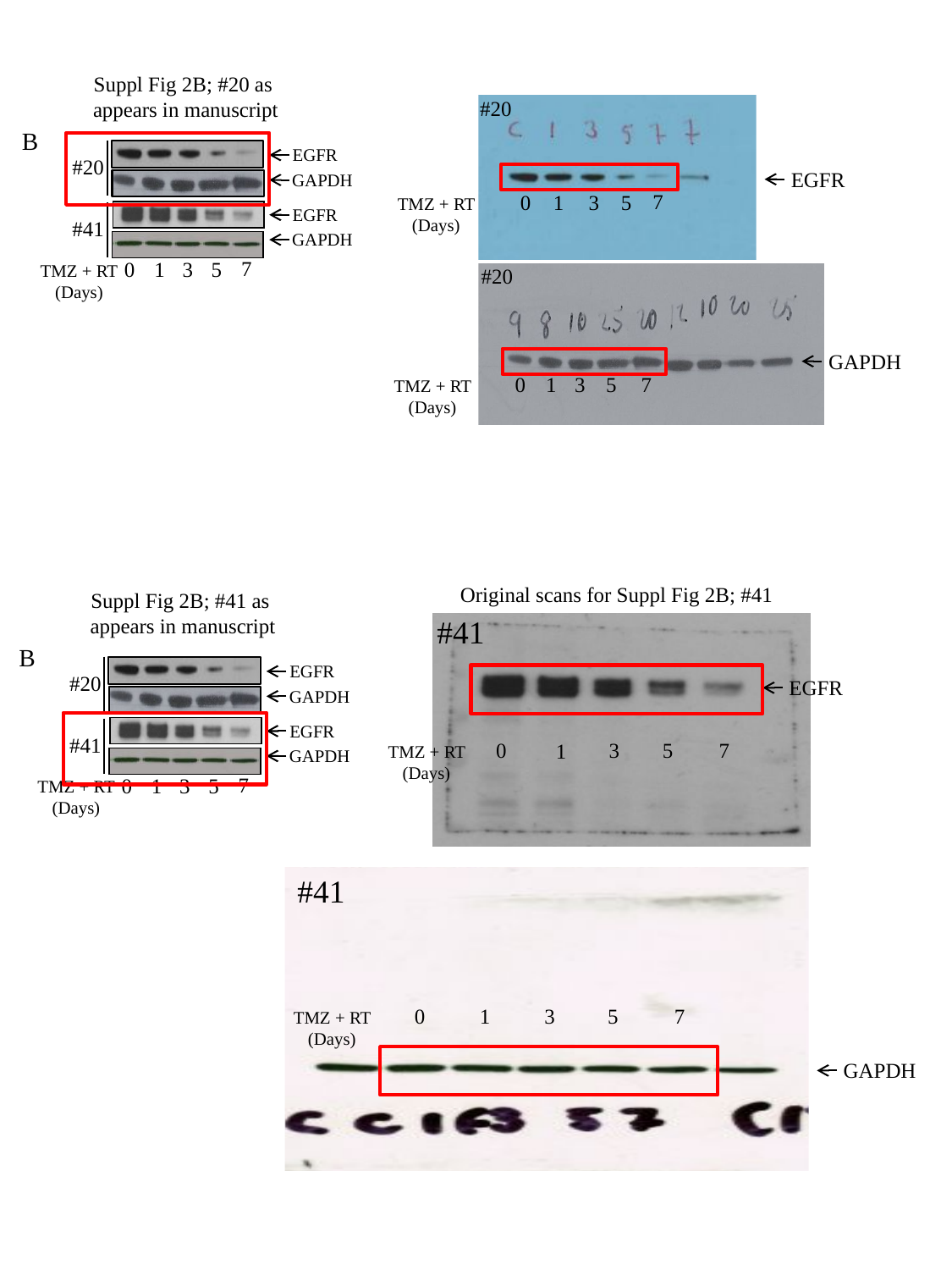

Suppl Fig 2B; #20 as
appears in manuscript
#20
B
EGFR
#20
GAPDH
EGFR
#41
GAPDH
7
0
3
5
1
TMZ + RT
(Days)
EGFR
7
0
3
5
1
TMZ + RT (Days)
#20
GAPDH
7
0
3
5
1
TMZ + RT (Days)
Original scans for Suppl Fig 2B; #41
Suppl Fig 2B; #41 as
appears in manuscript
#41
B
EGFR
#20
GAPDH
EGFR
#41
GAPDH
7
0
3
5
1
TMZ + RT
(Days)
EGFR
7
0
3
5
1
TMZ + RT (Days)
#41
7
0
3
5
1
TMZ + RT (Days)
GAPDH
